# Exploring Public, Practitioner and Policymaker Perspectives of Unhealthy Lifestyle Factors in the Context of Socioeconomic Deprivation: A Qualitative Study

**DOI:** 10.1111/hex.70069

**Published:** 2024-10-24

**Authors:** Hamish M. E. Foster, Frances S. Mair, Catherine A. O'Donnell

**Affiliations:** ^1^ General Practice and Primary Care, School of Health and Wellbeing, College of Medical, Veterinary and Life Sciences University of Glasgow Glasgow Scotland

**Keywords:** health behaviour, health inequalities, lifestyle, qualitative, socioeconomic factors, unhealthy lifestyle factors

## Abstract

**Introduction:**

Unhealthy lifestyle factors, such as smoking, high alcohol intake, poor diet and physical inactivity, are key risk factors for premature mortality. How unhealthy lifestyle factors are viewed in the wider context of socioeconomic deprivation is rarely considered. Understanding key stakeholder views on lifestyle factors in the context of deprivation is critical to intervention development and reducing harm in more deprived populations. The aim of this study was to explore public, healthcare professional and policymaker views around unhealthy lifestyle factors in the context of deprivation. The aim was broad to facilitate iterative development of ideas, as the views of this wide range of stakeholders are rarely captured.

**Methods:**

Twenty‐five adult members of the public in Scotland took part in four focus groups between August 2022 and June 2023. Eighteen semi‐structured interviews were conducted with professionals: 12 primary‐care practitioners and 6 public‐health practitioners and policymakers. Reflexive thematic analysis was undertaken.

**Results:**

Four main themes were developed: (1) Evolving complexity of lifestyle factors – the number of lifestyle factors that adversely impact health has grown, with increasingly complex interactions, (2) Social determinants of lifestyle – numerous links were made between socioeconomic conditions and unhealthy lifestyle factors by all participants, (3) Poverty as a fundamental social determinant – poverty was identified as a core factor for unhealthy lifestyle factors, and (4) Agency versus structure in relation to lifestyle – individual agency to address lifestyle factors was limited by structural constraints. Among professionals, understanding the challenging social determinants of unhealthy lifestyle factors was countered by a desire to support healthy change in those affected by deprivation.

**Conclusion:**

Public and professional views around lifestyle highlight an evolving understanding of the new and growing number of lifestyle factors as well as the increasingly complex interplay between lifestyle factors. Views of the social determinants of lifestyle and structural limits to agency strengthen arguments for reduced emphasis on individual‐level responsibility for unhealthy lifestyle factors and for deeper integration of social determinants into lifestyle interventions. In addition to addressing poverty and socioeconomic inequalities directly, innovative policy, planning and legislation that incorporate wider approaches could tackle upstream determinants of numerous unhealthy lifestyle factors simultaneously.

**Patient or Public Contribution:**

Members of the public who participated in this study have made contributions by sharing their views and perspectives. The National Health Service Research Scotland Primary Care Patient and Public Involvement (NRS PPI) Group contributed to the development of this work. The NRS PPI Group was consulted as part of the preparatory work for H. M. E. F.'s doctoral thesis funding application. The findings of the qualitative work were presented to them, and they informed the interpretation of those findings and related work presented at conferences and public engagement events.

## 1 Introduction

Lifestyle factors such as smoking, high alcohol consumption, poor diet and physical inactivity are leading risk factors for noncommunicable diseases and premature death [1]. Other lifestyle factors that are emerging include sleep [2], sedentary behaviours [3], updated dietary measures [4] and markers of social connection [5, 6]. Often referred to as unhealthy lifestyle factors, these tend to cluster in individuals leading to strong associations with adverse health [7, 8].

Further, there is a socioeconomic gradient in both mortality and lifestyle factors: more deprived populations have higher mortality and prevalence of unhealthy lifestyle factors [8, 9]. However, health inequalities persist after controlling for unhealthy lifestyle factors [10]. Further, there is disproportionate harm in more deprived populations, as the combined effect of unhealthy lifestyle factors and deprivation together is greater than the sum of their individual effects [11, 12].

To maximise health benefits, interventions and policies could tackle a wider range of lifestyle factors by targeting support to more deprived populations. Although population‐level interventions can have disproportionate benefit among more deprived populations [13, 14], interventions proportionate to need at individual or community levels are rare [15, 16, 17]. One reason may be the tension in how interventions and policies are operationalised, namely, at the level of individuals, communities or the wider society [18]. Barriers to progress may also include a lack of integration of ‘upstream’ drivers in interventions [19].

One way of considering the drivers of unhealthy lifestyle factors is through the lens of structural or social determinants of health [16, 20]. The ability to change and sustain healthier living requires not only individual agency, the capacity of individuals to make lifestyle choices, but also wider social, economic and political environments that enable healthy living [21]. These wider ‘upstream’ conditions determine the extent to which individuals can modify behaviours and sustain healthier ways of living [22]. For example, financial and employment factors influence choices and opportunities [23]. However, lifestyle factors are often viewed as matters of individual responsibility and lifestyle interventions often aim to inform individuals why and how to make healthier choices [24, 25]. Even when interventions focus on upstream determinants initially, they often ‘drift’ downstream to rely on individual‐level motivation and responsibility [26, 27].

Three issues may contribute to the focus on and drift towards individual responsibility. First, commercial determinants of health mean that corporations are incentivised to perpetuate the paradigm that targeting individual responsibility is the optimal mechanism by which to reduce harm from their products (e.g., tobacco) [28]. Second, individual responsibility for lifestyle is often the predominant narrative in media [29, 30]. Third, decision‐makers often fail to recognise lifestyle factors as social practices embedded in peoples' lives, social networks and local contexts that facilitate and limit choices. Similarly, shared characteristics of communities, which affect their ‘risk of risks’, are often viewed as distantly related to lifestyle factors [31]. For example, poverty and racism are shared experiences for communities and act as ‘fundamental causes’ of ill‐health, but are rarely addressed directly in lifestyle interventions [32, 33].

New understanding of how to optimise support for healthy living for those experiencing deprivation could be found: (1) in the growing range of lifestyle factors, (2) by considering several lifestyle factors simultaneously [34] and (3) via deeper integration of the social and structural determinants of health into interventions. Exploring stakeholders' views of these concepts could inform development of future lifestyle interventions [35].

This qualitative study aimed to explore the views of the public, healthcare professionals and policymakers in relation to unhealthy lifestyle factors in the context of socioeconomic deprivation. The aim was broad to facilitate higher level exploration of views around lifestyle factors generally and to deepen our understanding of how lifestyle factors are conceptualised.

Health is a devolved responsibility, with often clear policy and organisational differences between the UK's nations [17, 36]. For example, the smoking ban varied by nation, and minimum unit pricing for alcohol exists in Scotland but not in other UK nations [37, 38]. Therefore, we focussed on views of stakeholders in Scotland to provide more contextually relevant findings.

## 2 Methods

The work reported here was part of a wider mixed‐methods study with an overall aim of understanding the mortality associated with combinations of unhealthy lifestyle factors across the socioeconomic spectrum [39].

### 2.1 Study Design

This study used focus groups with members of the public and one‐to‐one semi‐structured interviews with professionals conducted between August 2022 and June 2023. Focus groups were chosen for public participants to gather a wider range of perspectives from a larger number of participants. One‐to‐one interviews were easier to organise with the professionals and permitted a more detailed exploration of their views [40].

### 2.2 Study Recruitment and Participants

Social media was used to recruit adult members of the public resident in Scotland. The study was advertised via social media channels, including those of third sector partners [41, 42]. Healthcare professionals (‘practitioners’) were recruited via professional networks [43]. Policymakers and public health professionals (PPHPs) were recruited via email using snowball sampling, professional networks and relevant organisations [44]. To be eligible, professional participants had to be working in Scotland and either provide face‐to‐face lifestyle advice or have relevant public health or policy‐making experience.

### 2.3 Data Collection

Three focus groups and all interviews were conducted online via video conferencing software. One focus group took place face‐to‐face. Focus groups lasted approximately 60–80 min; interviews lasted up to 60 min. Focus group guides and interview schedules (Appendix S1) were developed iteratively from findings from the project's related studies [11, 12, 45, 46], review of pertinent literature and the knowledge and interests of the study team (two academic general practitioners and a non‐clinical scientist with an interest in health inequalities). H. M. E. F. conducted all focus groups and interviews, supported by C. A. O. for three interviews and one focus group.

For public participants, age, gender, address and occupation were collected. Postcode of residence determined participants' Scottish Index of Multiple Deprivation score (https://simd.scot/), reported as 1st to 10th (most to least deprived) decile [47]. For professionals, data on gender and occupation were collected.

### 2.4 Data Analysis

Audio data were transcribed, anonymised and analysed using reflexive thematic analysis [48, 49]. A reflexive approach was chosen, as it provided flexibility when analysing the pre‐defined wide range of topics. An interpretative approach to analysis facilitated the researchers' use of their prior understanding, which was informed by the wider project and their varying fields of expertise [11, 12, 45, 46, 49].

Transcripts were imported into NVivo 14 to aid analysis. H. M. E. F. read all transcripts in detail, with C. O. D. and F. S. M. reading a selection of interviews. All researchers noted codes in the data and held regular analysis sessions to discuss the codes. Codes were grouped into common themes and later stages of coding refinement included discussing coding and interpretation with an advisory board and a PPI group.

## 3 Results

### 3.1 Participant Characteristics

Data were collected from 25 members of the public in four focus groups. Participants were aged between 24 and 78 years; 18 were men and 7 were women. A wide range of occupations was represented (Table 1). Participants tended to be from more affluent postcodes, but all deprivation deciles 1–10 were represented.

**Table 1:** Characteristics of focus group participants (members of the public).

Focus group	Online/face to face	*n*	Number female	Age range (years)	Occupation	SIMD decile
1	Online	6	1	25–36	Truck Driver, Social researcher, Accountant, Trader, Private teacher, Data Analyst	10, 4, 7, 3, 10, 10
2	Online	8	1	24–61	Plumber, Driver, Engagement Officer, Cashier, Teacher, Fashion Designer, Administrative officer, Farmer	10, 2, 2, 2, 5, 8, 5, 5
3	Online	7	2	24–30	Part‐time labourer, Part‐time worker, Student, Storekeeper, Part‐time database administrator, Between jobs (previously customer service manager), Part‐time information technology assistant	3, 8, *, 8, 2, 1, 9
4	Face‐to‐face	4	3	58–78	Project Manager, Retired civil service, Retired mental health programme manager, Retired Police Inspector	6, 10, 2, 10

*Note:* SIMD, Scottish Index of Multiple Deprivation 2020 (1 = most deprived decile, 10 = least deprived decile).

* Missing.

Semi‐structured interview data were collected from 18 professionals (six community links workers [CLWs: non‐clinical practitioners based in primary care, often in ‘socio‐economically deprived’ communities, who work with patients to navigate services] [50], one community nurse, one community pharmacist, four general practitioners [GPs] and six PPHPs).

### 3.2 Themes

Qualitative data are presented in relation to four generated themes (Table 2). Example quotes are provided verbatim. The corresponding participant type, gender (F/M) and, for public participants, age in years and SIMD decile (e.g., SIMD10 = 10th decile = least deprived) are given in parentheses following each quote.

**Table 2:** Themes.

1. Evolving complexity of lifestyle factors
2. The social determinants of lifestyle
3. Poverty as a fundamental determinant of lifestyle Subthemes – Direct impact of poverty on lifestyle; Indirect impact of poverty on lifestyle
4. Agency versus structure in relation to lifestyle factors

#### 3.2.1 Theme 1 – Evolving Complexity of Lifestyle Factors

All participants described a wide array of unhealthy lifestyle factors, evolving over time to become more complex. Public participants often referred to ‘conventional’ lifestyle factors such as smoking and diet, but also described a broad range of factors, including social media use, use of digital technology/smartphones and sleep. Public participants frequently suggested direct links between unhealthy lifestyle factors and social circumstances (e.g., shift work affecting sleep), but also characterised wider societal changes such as new technology or increasing levels of loneliness as unhealthy lifestyle factors themselves.

I think loneliness has a big impact, and I think it's getting worse, because there's a lot more single people.

(Public participant, 58F, SIMD6)

Practitioners also recognised a wide range of unhealthy lifestyle factors and described a shift from a narrow focus on more ‘traditional’ factors like smoking, high alcohol consumption, poor diet and physical inactivity to a much wider concept encompassing anything that people do regularly that could influence their health.

Yeah. I mean I think the very traditional … maybe the bit that I would have said if I was ten years ago is, yeah, things about diet, activity, which I would have called exercise, smoking, alcohol, drugs, I may or may not have mentioned, but I think those are the core bits.

(GP1, F)

PPHPs had the broadest view of unhealthy lifestyle factors, discussing them in terms of both individual behaviours and upstream social drivers that influenced opportunities.

So when I think about health behaviours, I think actually about what's driving those behaviours and the environment that are leading them to do that.

(PPHP5, M)

The growing complexity meant an expansion of the number of unhealthy lifestyle factors and including more facets of daily living. The language used had also evolved. Practitioners described how ‘exercise’ was now referred to as ‘physical activity’, with awareness that physical activity in a broader sense was associated with health benefits. CLWs used language such as ‘social connection’, highlighting awareness of broader and newer concepts that encompass social isolation and loneliness. Public participants also portrayed awareness of more ‘up‐to‐date’ terminology (e.g., ‘excessive alcohol consumption’ as opposed to ‘drinking’).

Yeah, you know, I would say, negatively, you know, I wouldn't just say, drinking of alcohol, yeah, I would use the word, excessive consumption of alcohol.

(Public participant, 26F, SIMD8)

For practitioners, the new terms helped them consider what might be feasible in terms of healthy change. For CLWs, this paralleled a critical appreciation of social circumstances as they weighed up what change was feasible, given the wider social context. CLWs addressed practical considerations as well as individuals' perceptions of what was achievable while using a language of broader and more nuanced descriptions of unhealthy lifestyle factors.

…we really want to encourage people to become more active. And […] something is better than nothing, you know, like just start? Okay two minutes, it's fine, it's two minutes more than you were doing before … people are coming from a baseline of nothing.

(CLW2, F)

There was also a complex interplay between lifestyle factors. Public participants recognised that lifestyle factors impact one another (e.g., screen time affecting sleep) but also that multiple unhealthy lifestyle factors increased the risk to health. For practitioners, lifestyle factors were rarely considered in isolation, and they viewed some factors as combined with others. For example, smoking, alcohol consumption and socialising were described as a triad, whereby improving one factor could impact on another. Likewise, discussions around sleep led to consideration of other factors that impact sleep, such as screen time and caffeine/alcohol consumption.

You will get people who will say, I can't do this because of that, and I can't do that because of this … Like, if I don't drink, I can't see my friends, because you only socialise through the pub. Or if I stop smoking, I can't see my friends because then I can't go for a drink, because if I do, I'll want to have a cigarette.

(Community nurse, F)

PPHPs and policymakers also described lifestyle factor interplay at the population level. Considering shared drivers for numerous unhealthy lifestyle factors, PPHPs suggested that several factors could be addressed simultaneously via consideration of health and well‐being more broadly at the level of communities rather than via specific unhealthy lifestyle factors enacted by individuals.

What do you need in order to be healthy, and how do you mitigate some of these unhealthy influences in order that they [unhealthy lifestyle factors] don't cluster because there's nothing else in communities?

(PPHP1, F)

#### 3.2.2 Theme 2 – The Social Determinants of Lifestyle

All participants saw difficult socioeconomic circumstances as a direct challenge to healthy ways of living. From the financial cost of a healthy diet to transport to access services, and proximity to green space, participants drew numerous detailed links between wider social circumstances and unhealthy lifestyle factors. Perceptions of inextricable pathways from socioeconomic conditions to unhealthy lifestyle factors were voiced from the outset and some participants' definitions of lifestyle factors were indistinguishable from socioeconomic factors. For example, public participants described education and the wider living environment as examples of unhealthy lifestyle factors, which are not generally considered as such by healthcare professionals.

So, I think it's everything, exercise, what you eat, drinking, smoking, drugs, just your family history. And how you've been taught about cooking, and what education you've had. And just what kind of stress you're under, and the environment that you live in, I think.

(Public Participant, 58F, SIMD6)

There was also a distinction between perceptions of the public and practitioners with those of PPHPs. Public participants and practitioners more readily discussed unhealthy lifestyle factors at the level of individual action. In contrast, PPHPs consistently took a broader view and were reluctant to use the terms ‘lifestyle factor’ or ‘health behaviour’. Instead, PPHPs referenced what they considered to be upstream drivers. ‘Lifestyle’ or ‘health behaviour’ were seen as unhelpful terms that referred to downstream proxies that drew attention away from upstream drivers.

I suppose there's also a general sense that the behaviour sits within a wider context of other things, that affect your health, and so I suppose, one of the things that I'm keen to do, is not overtly focus on the behaviour, at the expense of all the other things, that wider context that that behaviour is occurring in, that is also a factor in the outcome of poor health, whatever.

(PPHP1, F)

A focus on downstream lifestyle factors was perceived as shifting blame to individuals and risked both vilifying people in difficult circumstances and increasing health inequalities. PPHPs saw unhealthy lifestyle factors and the choices that individuals make as inseparable from their social circumstances. The value of focussing on upstream drivers of unhealthy lifestyle factors was further apparent in PPHPs' feelings of frustration by the siloing of funding and legal frameworks around unhealthy lifestyle factors (e.g., tobacco legislation, alcohol or fast‐food licencing, local council planning regulations) that typically follow ‘traditional’ lifestyle factor boundaries. This was viewed as reducing opportunities to address healthy living more generally.

So alcohol licensing sits apart, tobacco licensing sits apart and the regulation of fast food premises and so on. What we haven't been able to do, has been to join those up and think, alright, well actually what does a healthy community look like, what would you have?

(PPHP1, F)

#### 3.2.3 Theme 3 – Poverty as a Fundamental Determinant of Lifestyle

Poverty was explicitly expressed as an all‐encompassing factor that brought both direct and indirect challenges to healthy living.

##### 3.2.3.1 Theme 3 Subtheme – Direct Impact of Poverty on Lifestyle

The impact of poverty was often characterised as a straightforward lack of money that prevented access to healthy options.

Sometimes I barely eat a fresh meal, because I can't afford it. So, that has really restrained me to get some healthy diet, to take good care of myself, to live a kind of happy life, and get whatever I want.

(Public participant, 24M, SIMD3)

Connections were made between poverty and conventional factors but also between poverty and emerging factors. However, poverty meant not only a lack of resources that curtailed healthy lifestyle opportunities but also a lack of time and ‘head space’ to consider healthier options. This could even make an unhealthy choice the rational choice, given the circumstances.

If you're a single parent, you know, managing the household and children, dealing with the precariousness of poverty and insecure work. So the time to then think about eating healthily and looking after yourself and going and doing some exercise is really limited….

(PPHP4, F)

##### 3.2.3.2 Theme 3 Subtheme – Indirect Impact of Poverty on Lifestyle

Although poverty was perceived as a fundamental determinant and directly impacting unhealthy ways of living, poverty was also linked to unhealthy lifestyle factors indirectly via its impact on mental health. From a lack of money leading to anxiety, to benefits sanctions causing distress, or a lack of hope or sense of safety due to poor housing, all made unhealthy lifestyle factors more likely.

…when you're living below a certain income that you can't really afford everything and the pressure of taking care of your siblings, the pressure of providing for this, providing medical assistance, paying for groceries and all necessities, and mentally … the pressure can just develop anxiety….

(Public participant, 25M, SIMD10)

Mental health problems cited as due to or associated with poverty ranged from severe, such as drug or alcohol dependence following adverse childhood experiences, to less severe such as the ‘highs’ felt from sugary or processed food to ‘give you a kick in an otherwise quite bleak day’ (GP1, F). Links between adverse childhood experiences or psychological trauma and unhealthy lifestyle factors were widespread and perceived as a challenge to lifestyle interventions.

There's reasons why people can't, you know, aren't being physically active and everything, and it's not normally just a simple fix. Most of the people we see are extremely complex and quite often have that past trauma and it's, it is then, it takes it away from, you know, just the simple … yeah, let's have a wee plan and this is what we do and we work to that. Because of so much that they're dealing with….

(CLW3, F)

Unhealthy lifestyle factors were also seen as ‘coping mechanisms’ and as understandable responses to past experiences. Therefore, to make healthy lifestyle change, new coping mechanisms were required before people could address unhealthy lifestyle factors that they may be relying on.

…So like alcohol or smoking, can also offer respite from things that are happening in someone's environment too. […] Like, say, for example, if somebody has like high anxiety and smoking helps them with that, then it's helping their health in another way, but it may be kind of […] Like if they've got kids or if they drink a lot of alcohol and they have a stressful upbringing or something like that, a trauma response, then that can subdue that.

(Public participant, 26F, SIMD4)

Similarly, participants linked the experience of discrimination in forms of racism, bullying and perceived stigma to unhealthy lifestyle factors. Unhealthy lifestyle factors were seen as providing ‘a safe space’ from discrimination.

…sometimes people pick up these lifestyles of smoking and drinking as a result of being depressed from things like this [racism]. They see unhealthy lifestyles as a jacket of comfort to wear to just get on. I think inclusiveness and elimination of racism will go a long way.

(Public Participant, 26M, SIMD10)

Poverty was also seen to lower expectations for health and reduce motivation for healthy lifestyle change.

So it's no coincidence that people in their sixties are walking round with sticks in certain areas, whereas in other areas they're not. We can all see that, but I guess the expectation about health and what people can accept, to a certain degree, is maybe influenced by what we can see around us. And I guess that's from little kids, right up. If you're used to seeing people in their sixties, with high levels of disability, it's not so shocking if that happens to you.

(CLW2, F)

Poverty was thus a key driver that not only directly led to unhealthy lifestyle factors through a lack of resources but also indirectly by exacerbating a host of other factors that negatively influenced lifestyle.

#### 3.2.4 Theme 4 – Agency Versus Structure in Relation to Lifestyle Factors

Despite detailed descriptions of how challenging social circumstances made healthy ways of living difficult, most participants felt that individuals maintain some level of agency with which to make healthy choices. However, the opportunities for healthy choices and the level of agency to make those choices were seen on a spectrum contingent on wider social circumstances.

Even people who have been in the depths of substance use, can go on and make an amazing recovery, and live some of the healthiest lifestyles.

(CLW2, F)

Awareness of structural challenges to agency around unhealthy lifestyle factors generated a tension or dissonance, particularly among practitioners and PPHPs, as they estimated the level of agency to be accorded to individuals considering the structural challenges that they knew they faced in daily life.

So it's like having that awareness of if somebody's using a food bank, they don't have that freedom of choice to make those healthy decisions, they're basically just surviving. So how do you balance that?

(CLW4, F)

Among practitioners, this tension manifested as a balancing act between their understanding of the social determinants of unhealthy lifestyle factors and trying to support and improve the health and well‐being of individual patients. On the one hand, there was an appreciation of the lack of opportunities for healthy living, whereas on the other, there was a strong sense of duty to instil hope that healthy change was possible and to cultivate any agency despite difficult circumstances.

…I think we need to not do that either, to give up on people, because often folk will sense that and then feel that they feel that I've got no capacity to change … I think we have to, you know, believe in people's potential to change but just be realistic about how we enable people to do that.

(GP4, F)

For PPHPs, although the role of the social determinants of unhealthy lifestyle factors dominated their views, an apparent tension between agency and structure remained. This was expressed in terms of PPHPs' concerns of a disproportionate focus on helping people already living with unhealthy lifestyle factors (i.e., supporting the agency of individuals to make healthy choices) at the expense of wider prevention efforts to address the structural barriers to healthy living and a desire to redress that balance.

You need both prevention and the cessation, so we can't just prevent our way out of smoking and we can't just write off these tens of thousands of people who are smoking and basically leave them to die, so we have to help them…. So it's not a one prevention is good, upstream is good, downstream is bad, that actually you do need to have both of them.

(PPHP5, M)

## 4 Discussion

Key stakeholders' perspectives on unhealthy lifestyle factors in the context of socioeconomic deprivation include an evolving complexity of unhealthy lifestyle factors, with a greater and wider number of health and social factors considered ‘unhealthy lifestyle factors’, a greater appreciation of interactions between lifestyle factors and their understanding of the influence of socioeconomic conditions. Poverty emerged as a fundamental determinant in shaping both the prevalence of unhealthy lifestyle factors and the ability to enact and sustain healthy lifestyle change. The role of individual agency was viewed within structural constraints, leading to tensions between a desire to support individuals lead healthier lives while recognising the limits posed by wider structural forces.

### 4.1 Comparison With the Literature

The expansion of unhealthy lifestyle factors over time from a few specific ‘traditional’ lifestyle factors to a wider range mirrors the changes observed in the epidemiological literature [6, 51]. Among practitioners, this paralleled an increasingly nuanced exploration of people's social circumstances to support healthy change, highlighting a growing understanding of the role of social determinants in lifestyle [21, 32, 33].

The perceptions of the role of social determinants and of individual agency for unhealthy lifestyle factors are important to discuss in view of the reliance on individual responsibility in interventions [24]. All participants recognised limits to healthy living outside the control of individuals while also accepting a role for individual responsibility. Views captured here suggest appreciation of the influence of social contexts on lifestyle, including among members of the public, in contrast to dominant media portrayals [30]. A qualitative study exploring public views around food choice found numerous examples of an ‘illusion of food choice’, especially in difficult social circumstances [52]. However, the same study also highlighted a range of views on individual responsibility for healthy eating.

Practitioners' views in this study straddled both individual responsibility and wider social influences with a sense of duty to support individuals balanced by an understanding of the wider social context. PPHP views aligned with a greater role for social determinants, as they expressed a need to shift the focus from individual‐level lifestyle factors. The discrepancy of views between professional types is likely to be linked to their professional perspectives: individual versus population. A distinction between ‘sick individuals and sick populations’ is seen here in the perceptions of professionals dealing with unhealthy lifestyle factors of individuals versus those addressing unhealthy lifestyle factors of populations [53].

A recent qualitative study of GPs discussing obesity found that, compared to those working in more affluent areas, those working in more deprived areas were more likely to ascribe patients responsibility for their obesity [54]. The authors suggest that more challenging working conditions, greater ‘empathy fatigue’ and more frequent experiences of treatment failure among those working in more deprived areas resulted in feelings of frustration and blaming patients for their obesity. This was not apparent in this study, despite all the GPs and most of the other practitioners working in areas of high deprivation. However, practitioners in this study acknowledged both individual‐level responsibility and social circumstances as influences over unhealthy lifestyle factors. Further, desire to support individuals and instil hope at the same time as appreciating the structural limits to life chances resonates with a similar finding of ‘contradictory’ beliefs in an ethnographic study of community workers [55].

Participants' views of the greater role of social determinants fit with the health lifestyle theory conceptualised by Cockerham [56]. Cockerham develops previous sociological theories to describe a paradigm where ‘life choices (agency)’ and ‘life chances (structure)’ interact to influence individuals' ‘dispositions to act (habitus)’ and explain unhealthy lifestyle factors as wider social practices. Cockerham also describes sociological legacies that have resulted in an overemphasis on individual choice rather than opportunities and develops health lifestyle theory with the aim to ‘bring structure back’ [56], whereas the desire to support individual‐level lifestyle change seen among practitioners and PPHPs here may contribute to ‘lifestyle drift’ – ‘the tendency for policy to start off recognizing the need for action on upstream social determinants of health inequalities only to drift downstream to focus largely on individual lifestyle factors' [24, 26]. Even terms or concepts such as ‘upstream driver’ or ‘social determinants’ could also contribute to lifestyle drift if they are perceived by decision‐makers as concerned with issues more distantly related to unhealthy lifestyle factors when, in fact, and in line with views seen here, they are much closer and causally linked.

### 4.2 Implications

The increasing complexity of unhealthy lifestyle factors – the increasing number, type, definition/concept and interactions between lifestyle factors – suggests that practitioners could benefit from increased resources and training on supporting individuals with healthy living. The perceived causal influence of social determinants for unhealthy lifestyle factors means that greater integration of social determinants of lifestyle in interventions could be acceptable [19]. For example, perceived racism is associated with unhealthy lifestyle factors but rarely acknowledged in interventions [57, 58, 59].

One way of greater integration of social determinants into lifestyle interventions might be in considering the conscious workload required by an intervention. Interventions targeting conscious processes (e.g., providing risk information) require conscious effort from individuals to enact change compared with those that target non‐conscious processes (e.g., taxation) [60]. Highlighting conscious processes involved in interventions could help policymakers and researchers consider the resources required of individuals to make and sustain healthy lifestyle changes. This could help mitigate against the risk of exacerbating health inequalities via interventions that target conscious processes, as these ‘make higher demands on people's cognitive, social, and material resources’ [60]. For example, the lack of time and head space seen in this study's characterisation of poverty is consistent with lower conscious processing resources in communities experiencing deprivation and explains how an intervention might exacerbate inequalities. Similarly, highlighting the work required to lead a healthy life offers a way of viewing and supporting unhealthy lifestyle factors that could account for the social determinants of lifestyle [61, 62]. Alternatively, and consistent with how participants viewed the interrelationships between social circumstances and lifestyle, Frohlich et al. suggest studying the relationships between agency, lifestyle and structure to provide deeper understanding of what health inequalities are and propose an idea of ‘collective lifestyles’ that can bridge structure and agency and explain unhealthy lifestyles [63].

Future research could examine whether and how practitioners balance their desire to support healthy change with their appreciation of the structural determinants of lifestyle to inform practice. Examining for differential perceptions of lifestyle factors across socioeconomic groups, ascertaining how perceptions are formed, the influence of media and the ‘accuracy’ of perceptions would also be important future research topics. For policy, perceptions here support focussing on broader concepts of healthy living and rethinking the siloing of legislation, policies and funding by lifestyle factors.

### 4.3 Strengths and Limitations

This study captures perspectives from a wide range of stakeholders. A purposive sample of public participants ensured that a range of occupational types and deprivation levels were represented. SIMD is an area‐based measure that may misclassify individuals' level of deprivation. Public participants tended to be from more affluent postcodes relative to the Scottish general population, but all deciles were covered. Data on ethnicity were not collected. Therefore, the views of members of the public and resultant themes may not be generalisable. Findings may have more relevance for Scottish contexts given. Nevertheless, the broad nature of themes generated is likely to be of relevance in other settings.

## 5 Conclusion

Public and professional views of unhealthy lifestyle factors in the context of socioeconomic deprivation delineate an evolving complexity and interplay of unhealthy lifestyle factors and their underlying socioeconomic drivers. Among practitioners, appreciation of the social determinants of unhealthy lifestyle factors coexisted with a sense of duty to instil hope for healthy lifestyle change in individuals affected by socioeconomic deprivation. However, perceptions across participants captured here diminish the role of individual‐level responsibility for healthy choices and support closer integration of social and behavioural determinants of health. Innovative policy, planning and legislation are required to incorporate wider approaches and tackle upstream determinants of numerous unhealthy lifestyle factors simultaneously.

## Author Contributions

**Hamish M.E. Foster:** conceptualisation, methodology, data curation, formal analysis, project administration, writing–original draft, and investigation, writing–review and editing. **Frances S. Mair:** conceptualisation, methodology, investigation, formal analysis, supervision, writing–review and editing. **Catherine A. O'Donnell:** conceptualisation, methodology, investigation, formal analysis, supervision, writing–review and editing.

## Ethics Statement

This study was approved by the University of Glasgow's College of Medical Veterinary and Life Sciences Ethics Committee (application number 200210156). Participants were informed that their contribution was voluntary, that their data would remain anonymous and that they could withdraw from the discussion or interview at any time. All participants provided written and verbal consent to the study and were offered reimbursement for travel and a shopping voucher (public participants) or a small fee (professional participants) in line with NIHR guidance https://www.nihr.ac.uk/documents/payment-guidance-for-researchers-and-professionals/27392.

## Conflicts of Interest

The authors declare no conflicts of interest.

## Supporting information

Supporting information.

## Data Availability

Transcripts of interviews and focus groups may be available on request to the corresponding author.
