# Supplementary material for: Exploring Public, Practitioner and Policymaker Perspectives of Unhealthy Lifestyle Factors in the Context of Socioeconomic Deprivation: A Qualitative Study
Source: Health Expect. 2024 Oct 24;27(5):e70069. doi: 10.1111/hex.70069 (PMC11500207; doi:10.1111/hex.70069)
Supplement: Supplementary file 1 — Supporting information. [file HEX-27-e70069-s001.docx]

# APPENDIX – Topic Guide for Focus Groups and Interview Schedule

## Topic Guide for Focus Groups v3 24.06.2022

How do the public and professionals view high risk health behaviour combinations in the context of deprivation? What are the barriers and facilitators to incorporating the new evidence into daily life, practice, and policy to support healthy living in deprived areas?

**Introduction**

- Recorded – I will tell you when recorded starts and finishes – delete audio file as soon as we have accurate written copy of the discussion
- Confidentiality – anonymised
- Ask for your verbal consent at the beginning of recording – if you could just say your name and that you are happy to be recorded
- Do you mind introducing yourself to the group?
- Why have you decided to be part of today’s discussion?

**Public views of high-risk health behaviour combinations**

- We are exploring people’s views around health behaviours and lifestyle factors and how they combine with social and financial situations to affect our health.
- In your view, what kinds of behaviours do you consider of when you think of health behaviours/lifestyle factors?
- smoking and alcohol well known – what other factors do you consider sleep, being socially active, TV/screen time/specific foods or drinks
- more than one these behaviours can increase risks of health problems (like a heart attack or stroke) – do you ever consider combined effects where one behaviour may increase the negative effect of another
- some very specific combinations of unhealthy behaviours (e.g., if someone regularly smoked + regularly drank large amounts of alcohol + regularly ate too much processed meat) could have higher risk to people’s health than other combinations
- if this were true how might this change how you think about unhealthy behaviours?

**Public views of high-risk lifestyle/behaviour combinations in the context of deprivation**

- can you talk about the link between financial circumstances and health behaviours?
- what are the biggest or most important influences on health behaviours?
- How much control do we as individuals have over health behaviours given life circumstances?
- (stress, lack of time, job affecting sleep, housing affecting sleep, shops nearby affecting diet)
- can you talk about the link between financial circumstances and health itself?
- how are these links recognised? E.g., with how people give advice about health behaviours (?GP/nurse)
- Research suggests that combinations of unhealthy health behaviours might have an unequal effect on people’s health, where people who live in more disadvantaged areas are affected much more. what do you think this means for your own views or efforts to live healthily.
- Discuss how you feel this should change how people are supported to live healthy lives in more disadvantaged areas?
- Similarly the same may apply to people who are socially isolated , health behaviours may affect them much more.

**Setting the scene for incorporating new evidence into daily life**

- Describe how people are currently advised or encouraged to live healthy lifestyles?
- Describe how people are currently supported regarding healthy living/lifestyles?
- How do you think wider social and financial circumstances are taken into account when people are being advised/supported to live healthy lives?
- We are developing a lifestyle score – for people like link worker/pharmacist/nurse/doctor to discuss numerous lifestyle factors (smoking, how much alcohol you drink, how much physical activity you do) that contribute to a score.
- score could show which behaviours having biggest impact and which healthy changes could reduce chances of health problems (eg heart attack or stroke).
- Score could take into account socioeconomic status and social isolation – disproportionate risk for more deprived/isolated – similar to assign
- Could a score like this help you?
- How? (prioritise/ guide/ focus)
- problems foreseen? (? overwhelming/ highlighting multiple failure/ highlighting need for change for which there is minimal support)
- How would people want to receive this information? (app/visual)
- Who best placed to collect the information – person themselves? In conjunction with someone else? Prior to appointment? Etc

**individual barriers and facilitators**

o Considering examples in your work what are biggest or most common barriers individuals face in terms of addressing combinations of unhealthy behaviours -

o And opposite perspective, what are the main things that help (or could help individuals) them live a healthy life

**community barriers and facilitators**

o what are the biggest barriers in the local the local community/neighbourhood in terms of living addressing combinations of unhealthy behaviours?

o And what are the local community things that make it easier to live a healthy life in your area

o If you could change one thing in your local community to make it easier to live in a healthy way..?

o Eg shops, parks, sports facilities, social activities

**society barriers and facilitators**

o what do you think are the barriers in wider society (not just in local area) that make it hard to hard to addressing combinations of unhealthy behaviours

o And what are the aspects of society that help (or could help if they were changed) us live in healthy ways

o Councils/governments/legislation (smoking and alcohol, sugar taxes etc)

o How should society change to help people with many unhealthy behaviours

## Interview Schedule for Health or Public Health practitioners & Policymakers

How do the public and professionals view high risk health behaviour combinations in the context of deprivation? What are the barriers and facilitators to incorporating the new evidence into daily life, practice, and policy to support healthy living in deprived areas?

**Introduction**

1. Please introduce yourself
2. Briefly describe your job/work
3. In more detail, can you describe the aspects of your work that involve supporting and advising patients/public to live healthy lives with respect to healthy lifestyle factors/behaviours (e.g., smoking, alcohol, physical activity etc.)?
4. Describe if and how that aspect of your work (supporting and advising on healthy living) considers wider social factors?

**Professionals’ views of high-risk lifestyle/behaviour combinations**

- Please explain what you understand of the risks associated with having multiple or a combination of unhealthy lifestyle factors? (prompts - risk of: death, CVD, cancer, mental health problems etc.)
- Describe any combinations of unhealthy lifestyle factors you feel incur the greatest health risks?

1. Our work suggests that some specific combinations of lifestyle factors are particularly high risk (e.g., smoking + high processed meat intake + social isolation)
2. How does this change your understanding of lifestyle factors and risk?
3. Would you, and how would you, incorporate such information into daily work/advice.
4. How might patients/public respond to this evidence/information

**Professionals’ views of high-risk combinations in context of deprivation**

1. How might the same risk factors, or combinations of factors, affect different groups differently? (Prompts: demographics – age, gender, ethnicity, socioeconomic status)
2. How health risks discussed above (combinations of lifestyle/behaviours) are influenced by socioeconomic status/factors?
3. Combinations of unhealthy lifestyle factors may have a disproportionate effect on more deprived populations - not just because there are more unhealthy behaviours present but because they are somehow more susceptible. Why would that be?
4. How do you feel a disproportionate effect would or should change your work in advising/supporting healthy living?
5. How do you feel this would or should change how patients/populations are supported to live healthily in more socioeconomic disadvantaged contexts?

**Individual level barriers and facilitators to incorporating new evidence from our work into daily life that could support healthy living in deprived areas.**

Considering the work that would be required of patients and populations to live free from high-risk lifestyle combinations:

1. Discuss potential barriers that might prevent or hamper patients (practitioners) or populations (public health/policy makers) from addressing combinations of unhealthy lifestyle factors considering:
   1. very high-risk combinations
   2. disproportionate lifestyle risks felt by more deprived groups
2. Discuss factors that could help or facilitate patients or populations from addressing combinations of unhealthy lifestyle factors considering:
   1. very high-risk combinations
   2. disproportionate lifestyle risks felt by more deprived groups

**Professionals’ views of barriers and facilitators to incorporating new evidence from our work into practice and policy to support healthy living in deprived areas?**

Considering what would be required to change how you currently work or to change your policy aims:

1. Discuss potential barriers that might prevent or hamper changing your work/policy to improve patients’ or populations’ health outcomes considering:
   1. very high-risk combinations
   2. disproportionate lifestyle risks felt by more deprived groups
2. Similarly, please discuss factors that could help or facilitate changing your work/policy to improve patients’ or populations’ health outcomes considering:
   1. very high-risk combinations
   2. disproportionate lifestyle risks felt by more deprived groups
3. Is there anything else you would like to add?

N.B. being a semi-structured interview, participants’ answers will determine the interview questions that are asked as follow-ups.
